# A novel and simple cardiac magnetic resonance score (PE^2^RT) predicts outcome in takotsubo syndrome

**DOI:** 10.1007/s00330-023-09543-x

**Published:** 2023-03-23

**Authors:** Alexander Isaak, Johanna Bratz, Dmitrij Kravchenko, Narine Mesropyan, Irina Eckardt, Leon M. Bischoff, Leonie Weinhold, Daniel Kuetting, Claus Christian Pieper, Ulrike Attenberger, Sebastian Zimmer, Julian A. Luetkens

**Affiliations:** 1 Department of Diagnostic and Interventional Radiology, University Hospital Bonn, Venusberg-Campus 1, 53127 Bonn, Germany; 2 Quantitative Imaging Lab Bonn (QILaB), University Hospital Bonn, Bonn, Germany; 3 Institute of Medical Biometry, Informatics, and Epidemiology, University Hospital Bonn, Bonn, Germany; 4 Department of Internal Medicine II - Cardiology, University Hospital Bonn, Bonn, Germany

**Keywords:** Takotsubo syndrome, Magnetic resonance imaging, Major adverse cardiac events, Complications, Patient outcome assessment

## Abstract

**Objectives:**

To find simple imaging-based features on cardiac magnetic resonance (CMR) that are associated with major adverse cardiovascular events (MACE) in takotsubo syndrome (TTS).

**Methods:**

Patients with TTS referred for CMR between 2007 and 2021 were retrospectively evaluated. Besides standard CMR analysis, commonly known complications of TTS based on expert knowledge were assessed and summarised via a newly developed PE^2^RT score (one point each for pleural effusion, pericardial effusion, right ventricular involvement, and ventricular thrombus). Clinical follow-up data was reviewed up to three years after discharge. The relationship between PE^2^RT features and the occurrence of MACE (cardiovascular death or new hospitalisation due to acute myocardial injury, arrhythmia, or chronic heart failure) was examined using Cox regression analysis and Kaplan–Meier estimator.

**Results:**

Seventy-nine patients (mean age, 68 ± 14 years; 72 women) with TTS were included. CMR was performed in a median of 4 days (IQR, 2–6) after symptom onset. Over a median follow-up of 13.3 months (IQR, 0.4–36.0), MACE occurred in 14/79 (18%) patients: re-hospitalisation due to acute symptoms (9/79, 11%) or chronic heart failure symptoms (4/79, 5%), and cardiac death (1/79, 1%). Patients with MACE had a higher PE^2^RT score (median [IQR], 2 [2–3] vs 1 [0–1]; *p* < 0.001). PE^2^RT score was associated with MACE on Cox regression analysis (hazard ratio per PE^2^RT feature, 2.44; 95%CI: 1.62–3.68; *p* < 0.001). Two or more PE^2^RT complications were strongly associated with the occurrence of MACE (log-rank *p* < 0.001).

**Conclusions:**

The introduced PE^2^RT complication score might enable an easy-to-assess outcome evaluation of TTS patients by CMR.

**Key Points:**

*• Complications like pericardial effusion, pleural effusion, right ventricular involvement, and ventricular thrombus (summarised as PE* ^*2*^ *RT features) are relatively common in takotsubo syndrome.*

*• The proposed PE* ^*2*^ *RT score (one point per complication) was associated with the occurrence of major adverse cardiac events on follow-up.*

*• Complications easily detected by cardiac magnetic resonance imaging can help clinicians derive long-term prognostic information on patients with takotsubo syndrome.*

## Introduction

Takotsubo syndrome (TTS), also known as broken heart syndrome or stress-induced cardiomyopathy, is an acute transient cardiomyopathy characterised by left ventricular (LV) systolic dysfunction with reversible regional wall motion abnormalities (RWMA) [1]. The apical segments are most commonly affected by circumferential RWMA (“apical ballooning”); however, diverse atypical forms including midventricular, basal, and focal types exist [2]. TTS is commonly associated with emotional or physical triggers and its clinical presentation often mimics acute coronary syndrome (ACS) [3].

While the clinical spectrum and epidemiological features of TTS have become increasingly well characterized in recent decades, the data on its long-term prognosis is conflicting: TTS was widely believed to be a benign disease with favourable prognosis and complete recovery of LV systolic function [4]. However, recent studies indicate that the long-term mortality rate of patients with TTS is much higher than expected and is comparable to or even exceeds that of patients with acute myocardial infarction [2, 5, 6]. Previous outcome studies demonstrated that clinical and imaging parameters such as male sex, age, LV ejection fraction, physical trigger, right ventricular (RV) involvement, and non-apical ballooning were factors related to worse long-term prognosis [5, 7–10].

Cardiac magnetic resonance (CMR) is an important tool in the diagnostic work-up of suspected TTS as it can confirm its diagnosis and differentiate it from other causes of acute cardiac symptoms, such as acute myocarditis [1, 11]. CMR features of TTS are typically circumferential RWMA with corresponding myocardial oedema and absent ischemic or inflammatory late gadolinium enhancement (LGE) in regions of abnormal function [12–14]. In addition, concomitant complications such as pericardial effusion, pleural effusion, RV involvement, or intraventricular thrombus, which have been described with poor outcomes [15–17], can be easily assessed on CMR. While the diagnostic utility of CMR in TTS is well established, its prognostic value remains poorly studied.

In this study, we explored the association between pathological CMR findings and the occurrence of long-term major adverse cardiovascular events (MACE) in TTS patients. In particular, easy-to-assess complications like the presence of pericardial and pleural effusions, RV involvement, or intraventricular thrombus, were analysed. The aim of the study was to find simple CMR imaging features associated with TTS outcomes that could be incorporated into an imaging-based score in the daily clinical routine of any cardiovascular imaging centre.

## Materials and methods

### Study population

This retrospective, observational single-centre study was conducted at a tertiary care centre. From November 2007 to January 2021, patients with clinical suspicion of TTS referred for CMR were identified. TTS was defined based on the International Takotsubo Diagnostic Criteria including CMR results and standard clinical work-up [18]. According to these recommendations, the main diagnostic criterion was the presence of (transient) left ventricular RWMA extending beyond a single coronary vascular distribution and presenting as apical ballooning or midventricular, basal, or focal wall motion abnormalities. Also, concomitant coronary artery disease was not a general contradiction for takotsubo syndrome. Patients with positive LGE who had typical RWMA and fulfilled diagnostic criteria for TTS were also included (e.g., RWMA beyond myocardial scarring) [18]. Patients with other CMR diagnosis were excluded (e.g., infectious myocarditis). Clinical characteristics were received from in-house medical records. The study complies with the principles of the Helsinki Declaration and was approved by the local institutional review board that waived informed consent due to retrospective study design (number: 303/16).

### Cardiac magnetic resonance

CMR was performed using 1.5-Tesla systems (1.5 Ingenia and 1.5 Intera, Philips Healthcare). For signal reception, a 16-channel body array coil with a digital interface was used. The CMR protocol consisted of electrocardiogram-gated steady-state free-precession cine imaging (four-chamber, two-chamber, short-axis, and LV outflow tract view) for functional analyses, T2-weighted short-tau inversion-recovery imaging (short-axis, two-chamber, and transversal view) for the assessment of myocardial oedema, and LGE imaging using segmented inversion-recovery gradient-echo sequence (short-axis, two-chamber, and four-chamber view) for the detection of myocardial scarring, necrosis or fibrosis. In a subgroup of patients, additional parametric mapping sequences were available (T1 mapping based on a standard 3(3)3(3)5 modified Look-Locker inversion recovery (MOLLI) acquisition scheme and T2 mapping based on a 6-echo gradient spin echo (GraSE) sequence). Mapping sequences were performed as previously described [14, 19, 20]. Contrast was given as a single bolus of 0.2 mmol/kg body weight of gadobutrol (Gadovist, Bayer Healthcare).

### CMR analysis

Images were analysed in consensus by two radiologists (J.A.L. with 10 years of CMR experience, A.I. with 5 years of CMR experience) using certified software (IntelliSpace Portal Version 12, Philips Medical System). Readers were blinded to clinical outcomes. Functional analysis (LV end-diastolic volume, ejection fraction, and longitudinal/circumferential/radial strain), assessment of myocardial oedema by visual and semi-quantitative approach (T2 signal intensity ratio), presence of myocardial scars by LGE imaging, and quantitative assessment of global myocardial T1 and T2 relaxation times, and haematocrit corrected extracellular volume fraction (ECV) values were performed standardised according to current recommendations including centre-specific reference values as previously described [19–22]. RWMA type was assessed regarding localisation: basal, midventricular, apical, or focal. Additionally, the presence of pleural effusion (> 20 mm) and pericardial effusion (> 5 mm), right ventricular involvement with visual RWMA [10], and ventricular thrombus was assessed and classified as “present” or “not present”. Based on expert knowledge, the PE^2^RT score was built by calculating the sum of these typical TTS complications (one point for each). The calculated PE^2^RT score ranged from 0 (no complication) to 4 (all defined complications present).

### Outcome assessment

Follow-up data were collected by reviewing in-house and outpatient medical records. MACE was defined as a composite of cardiovascular death, re-hospitalisation due to acute myocardial injury (e.g., positive troponin, electrocardiogram abnormalities, angina pectoris), new onset of arrhythmia, or re-hospitalisation due to chronic heart failure symptoms. Cardiovascular death was defined as death directly related to heart failure, myocardial infarction, arrhythmia, or cardiovascular disease. Other causes of death were classified as non-cardiovascular or unknown. The primary endpoint was the occurrence of MACE up to 3 years after discharge.

### Statistical analysis

Statistical analysis was performed using Prism (version 8.4.3; GraphPad Software Inc.) and SPSS Statistics (version 26; SPSS Inc., IBM). Normality assumptions were assessed by visual inspection of the data distribution supplemented by the Shapiro-Wilk test. For continuous variables, data are presented as means ± standard deviation or median with interquartile range (IQR) as appropriate. Categorical variables were presented as absolute frequencies with percentages in parentheses. Group comparisons between patients with and without MACE were performed using Student’s *t*-test or Mann-Whitney’s *U*-test for continuous variables, and chi-squared test or Fisher’s exact test for dichotomous variables. Univariable Cox regression analysis was applied to test the association of different clinical and imaging parameters regarding MACE. After a forward selection of relevant covariates with *p* < 0.05 in univariable analysis, covariates were added in a multivariable model to further fit the impact of variables. Kaplan-Meier analysis stratified by PE^2^RT score (< 2 vs.  ≥ 2) was performed, and a log-rank test was used to compare survival curves. The level of statistical significance was set to *p* < 0.05.

## Results

**Fig. 1:**
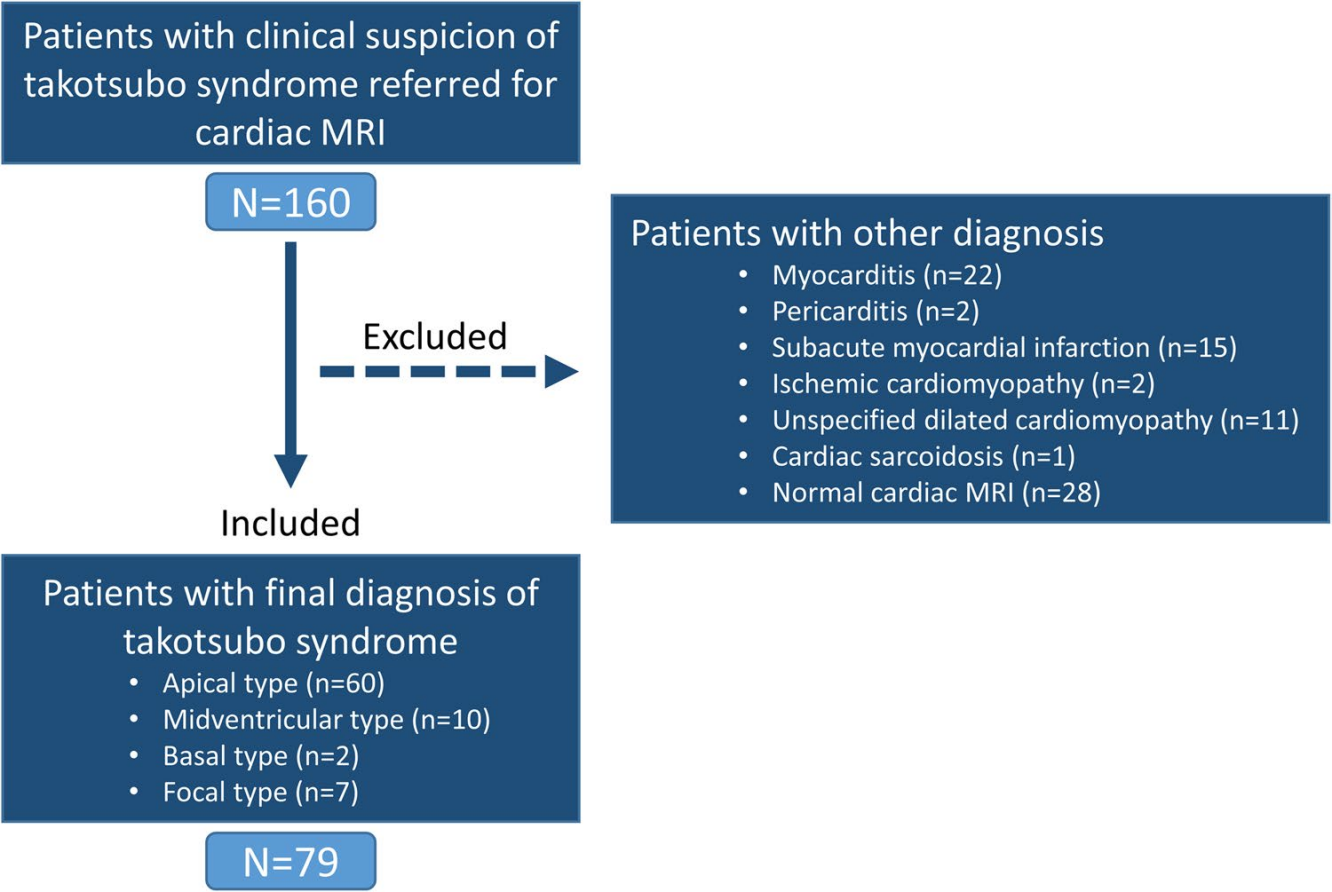
Study flow diagram

One-hundred-sixty patients referred for CMR with clinical suspicion of TTS were identified. Patients with unconfirmed TTS diagnosis were excluded (*n* = 81). Patients with a diagnosis of TTS (*n* = 79) were included (mean age, 68 ± 14 years; 72 women, 91%) for analysis. A study flow diagram is presented in Fig. 1.

### Clinical characteristics

**Fig. 2:**
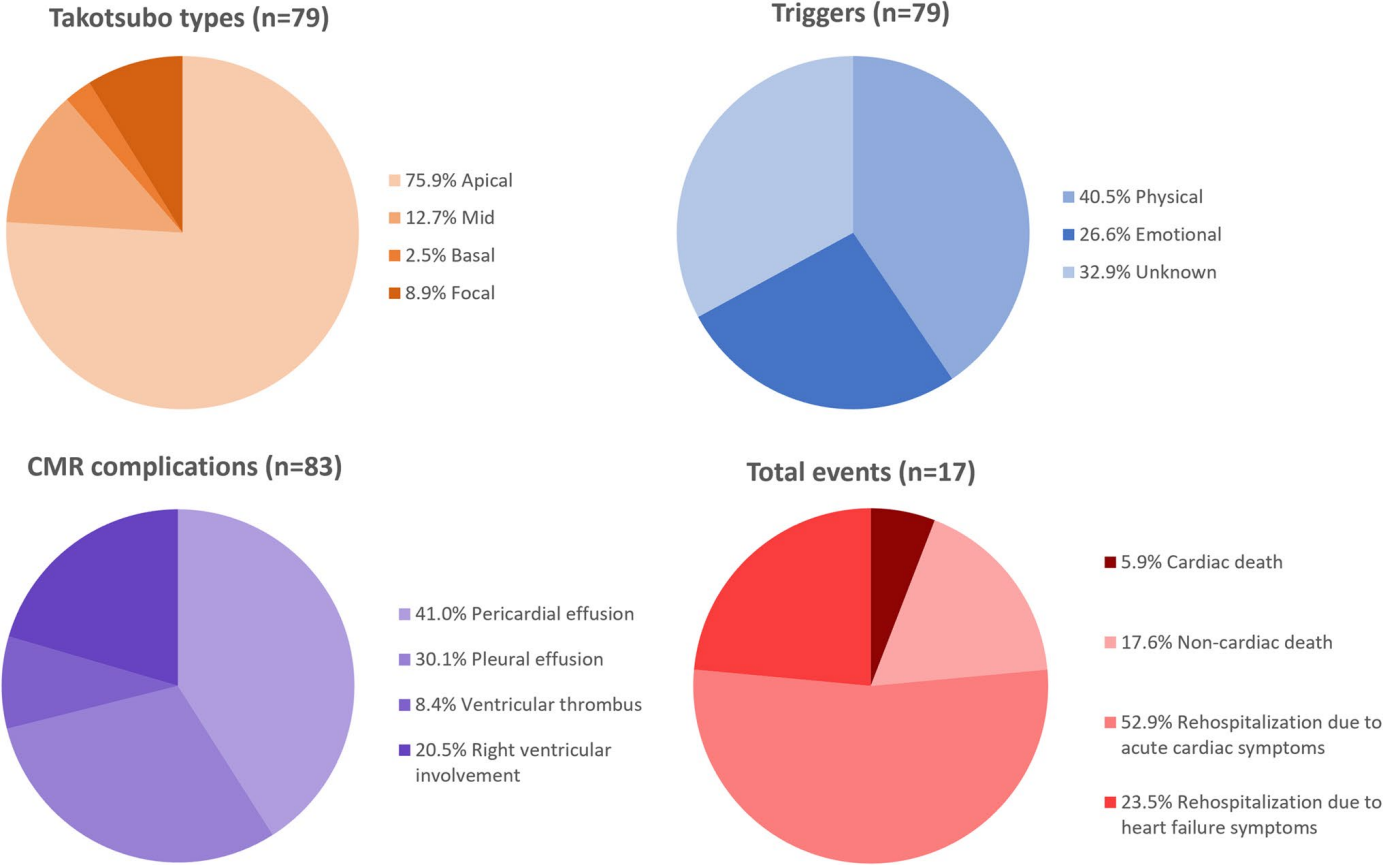
Parts of whole graphs show distribution according to types of takotsubo syndrome, preceding trigger factors, complications detected on cardiac magnetic resonance (CMR), and total events on long-term follow-up

The demographic and clinical data of the study population at the time of CMR are shown in Table 1. The most common symptoms on admission were chest pain (49/79, 62%), electrocardiogram abnormalities (47/79, 59%; ST-segment elevation/depression and/or T wave changes), and elevated troponin levels (75/79, 95%). In most patients (53/79, 67%) a preceding trigger event was identified: physical stress in 32/79 patients (41%) or emotional stress in 21/79 patients (27%) (Fig. 2). Coronary angiography was performed in 77/79 patients (97%) at the time of admission. Coronary heart disease was diagnosed in 31/77 of these patients (40%), and 5/77 of these patients (6%) needed coronary intervention due to coronary obstruction. However, the ventriculography and/or the clinical setting indicated co-existing TTS. Thus, these patients were additionally referred for CMR. All included patients with co-existing non-obstructive and obstructive coronary heart disease had CMR findings typical for TTS (circumferential RWMA extending beyond the territory of the involved coronary artery). Twenty-nine out of 79 patients (37%) were admitted to intermediate care or intensive care unit, with a median stay of 2 days (IQR 1–6). Six out of 79 patients (8%) developed cardiogenic shock during their stay.

**Table 1:** Clinical and cardiac magnetic resonance characteristics of patients with takotsubo syndrome according to the occurrence of major adverse cardiac events (MACE) during follow-up

Parameters	Total cohort (*n* = 79)	No MACE (*n* = 65)	MACE (*n* = 14)	*p* value
*Demographics and medical history*				
Age (years)	68 ± 14	68 ± 13	64 ± 18	0.47
Sex (female)	72 (91%)	59 (91%)	13 (93%)	0.80
Body mass index (kg/m^2^)	26 ± 5	26 ± 5	26 ± 5	0.83
Arterial hypertension	57 (72%)	44 (68%)	13 (93%)	0.057
Diabetes mellitus	17 (22%)	11 (17%)	6 (43%)	**0.032**
Hyperlipidemia	43 (54%)	35 (54%)	8 (57%)	0.82
Atrial fibrillation	10 (13%)	8 (12%)	2 (14%)	0.84
*Laboratory data*				
Elevated troponin T/I^*^	77 (98%)	63 (97%)	14 (100%)	0.51
Troponin T (ng/L)^*^	368 (159–629)	368 (159–550)	809 (104–1918)	0.48
Troponin I (ng/L)^*^	2.23 (0.84–4.82)	2.26 (0.96–4.97)	0.94 (0.12–4.30)	0.20
NT pro-BNP (pg/mL)^*^	4396 (1494–6560)	5778 (566–6978)	1948 (1804–2103)	0.43
CK-MB mass (ng/mL)^*^	9.6 (4.8–19.6)	10.8 (4.9–18.8)	6.9 (3.5–21.1)	0.36
White blood cell count (10^3^/µL)	10.8 (8.4–13.4)	10.5 (8.1–13.3)	11.8 (10.2–16.2)	0.19
C-reactive protein (mg/L)	11.0 (6.0–30.1)	11.2 (6.3–30.1)	10.2 (4.6–68.4)	0.72
*Clinical presentation on admission*				
Chest pain	49 (62%)	41 (64%)	9 (64%)	0.97
Dyspnea	39 (49%)	32 (50%)	7 (50%)	0.52
Acute electrocardiogram abnormalities^#^	47 (59%)	40 (63%)	7 (50%)	0.69
Physical trigger	32 (41%)	21 (33%)	11 (79%)	**0.001**
Emotional trigger	21 (27%)	19 (30%)	2 (14%)	0.25
Unknown trigger	26 (33%)	25 (39%)	1 (7%)	**0.02**
*Cardiac magnetic resonance*				
Heart rate (bpm)	72 ± 15	70 ± 13	82 ± 19	**0.004**
Left ventricular ejection fraction (%)	46 ± 13	48 ± 12	40 ± 14	**0.03**
Left ventricular end-diastolic volume index (mL/m^2^)	70 ± 17	69 ± 17	72 ± 15	0.44
Global longitudinal strain (%)	−10.8 ± 5.1	−10.3 ± 4.6	−13.0 ± 6.5	0.07
Global circumferential strain (%)	−15.6 ± 6.4	−15.1 ± 6.5	−17.5 ± 6.1	0.20
Global radial strain (%)	27.6 ± 15.3	26.5 ± 15.8	32.3 ± 11.9	0.19
Interventricular septal thickness (mm)	9.7 ± 1.7	9.9 ± 1.8	9.1 ± 1.5	0.10
Hypo- or akinetic wall segments (17-segment model)	9 (5–11)	9 (5–11)	8 (5–11)	0.79
Apical ballooning	60 (76%)	48 (75%)	12 (86%)	0.35
Mid RWMA	10 (13%)	9 (14%)	1 (7%)	0.49
Basal RWMA	2 (3%)	2 (3%)	0 (0%)	0.51
Focal RWMA	7 (9%)	6 (9%)	1 (7%)	0.80
Visible focal myocardial edema	74 (94%)	62 (97%)	12 (86%)	0.18
T2 signal intensity ratio	2.1 ± 0.5	2.2 ± 0.5	1.8 ± 0.3	**0.01**
Any late gadolinium enhancement	11 (14%)	9 (14%)	2 (14%)	0.97
Late gadolinium enhancement with ischemic localisation	5 (6%)	3 (5%)	2 (14%)	0.18
T1 relaxation time, native (msec)^†^	1099 ± 57	1102 ± 54	1083 ± 67	0.43
T2 relaxation time (msec)^†^	67 ± 10	68 ± 10	66 ± 10	0.66
Extracellular volume fraction (%)^†^	32 ± 6	32 ± 6	34 ± 8	0.47
Pericardial effusion	34 (43%)	22 (34%)	12 (86%)	** < 0.001**
Pleural effusion	25 (32%)	15 (23%)	10 (71%)	** < 0.001**
Ventricular thrombus	7 (9%)	3 (5%)	4 (29%)	**0.004**
Right ventricular involvement	17 (22%)	9 (14%)	8 (57%)	** < 0.001**
PE^2^RT score = 0	30 (38%)	30 (46%)	0 (0%)	**0.001**
PE^2^RT score ≥ 1	49 (62%)	35 (54%)	14 (100%)	**0.001**
PE^2^RT score ≥ 2	21 (27%)	9 (14%)	12 (86%)	** < 0.001**
PE^2^RT score ≥ 3	9 (11%)	4 (6%)	5 (36%)	**0.002**
PE^2^RT score (0–4)	1 (0–2)	1 (0–1)	2 (2–3)	** < 0.001**

*p* values are given for comparison between patients with and without major adverse cardiac events (MACE). Bold values denote statisticial significance (*p* < 0.05). *PE*^*2*^*RT score*, pleural effusion, pericardial effusion, ventricular thrombus, right ventricular involvement (each complication accounting for 1 point); *NT pro-BNP*, N-terminal pro-B-type natriuretic peptide; *CK-MB mass*, creatin-kinase muscle-brain type; *RWMA*, regional wall motion abnormalities

^*^ Laboratory data was available as follows: NT pro-BNP was available in 11/79 patients (14%), CK MB was available in 64/79 patients (81%), troponin T was available in 19/79 patients (24%), troponin I was available in 60/79 patients (76%). Elevated troponin was defined as troponin I  ≥0.05 ng/L and troponin T  ≥14 ng/L

^†^ T1 / T2 mapping, and ECV were available in 38/79 patients (48%)

^#^ ST-segment elevation/depression, T wave changes

### CMR characteristics

**Fig. 3:**
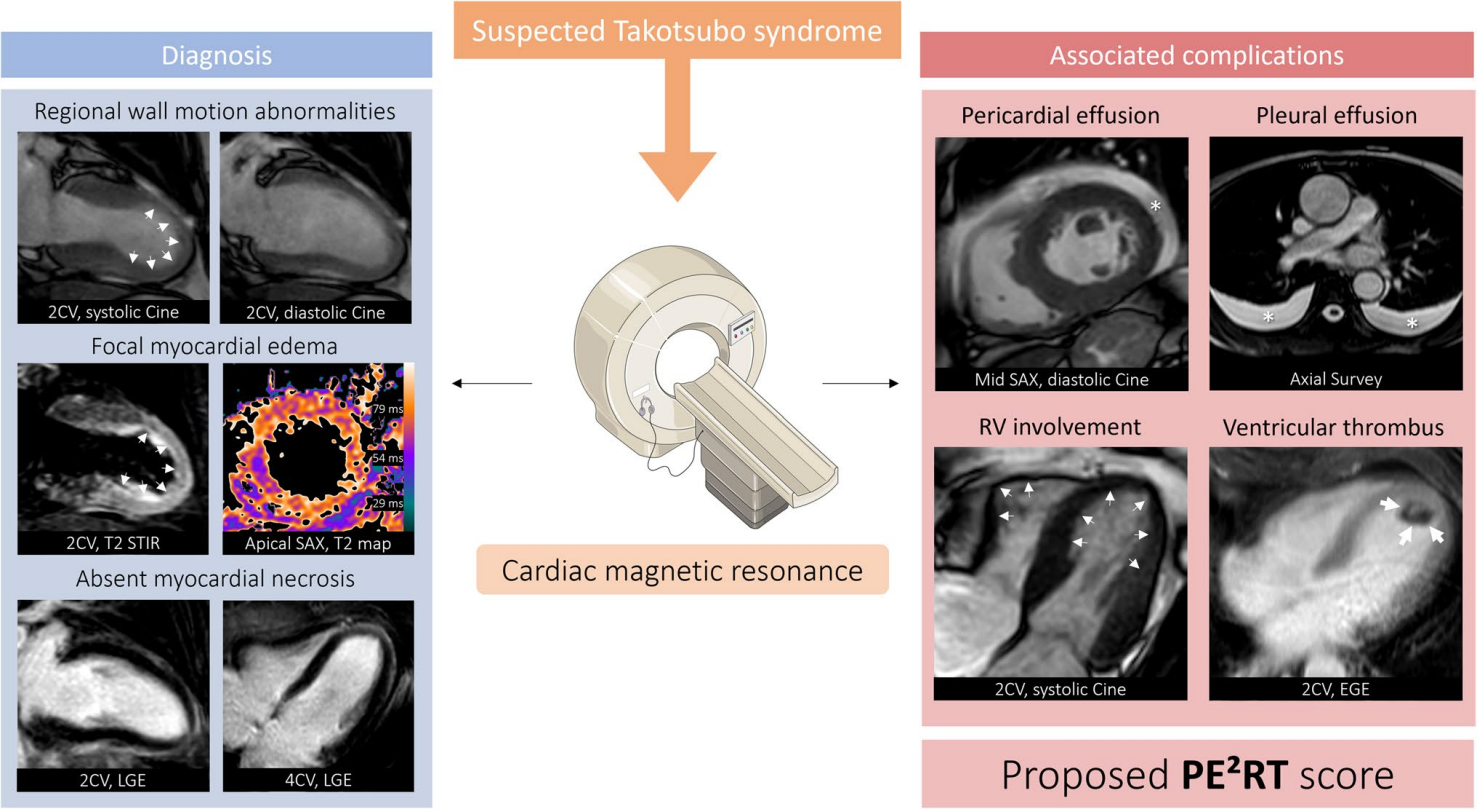
Figure composition shows the combined role of cardiac magnetic resonance for establishing the diagnosis of takotsubo syndrome and assessment of associated complications. Proposed PE^2^RT complications are illustrated: Pericardial effusion, pleural effusion, right ventricular (RV) involvement, ventricular thrombus (score calculated from the sum of complications, ranging from 0 to 4). 2CV, two-chamber view; 4CV, four-chamber view; SAX: short-axis view; STIR: short-tau inversion recovery; LGE, late gadolinium enhancement; EGE, early gadolinium enhancement. The figure contains modified free medical images from Servier Medical Art (https://smart.servier.com) under a CC BY 3.0 license

The CMR characteristics of the study population are given in Table 1. CMR was performed a median of 4 days (IQR 2–6) after symptom onset. TTS patients had reduced LV ejection fraction (46 ± 13%). Forty-seven out of 79 patients (59%) had an LV ejection fraction below 50%. The apical TTS type was most frequently observed (60/79, 76%). According to the 17-segment model, a median of 9 segments (IQR, 5–11) were hypokinetic or akinetic. The majority of patients showed visual myocardial oedema corresponding to the RWMA (74/79, 94%). Mapping parameters were available in 37/79 patients (47%). Based on the local reference value for inflammatory cardiomyopathy, global T1 relaxation times were elevated in all patients (37/37, 100%), and global T2 relaxation times in 36/37 patients (97%). LGE lesions were present in 11/79 patients (14%; 5/79 [6%] with ischemic, and 6/79 [8%] with non-ischemic distribution) and were unrelated to RWMA and myocardial oedema. Pericardial effusion (34/79, 43%) and pleural effusion (25/79, 32%) were the most frequently associated complications. RV involvement was seen in 17/79 patients (22%) and intraventricular thrombus in 7/79 patients (9%). The PE^2^RT score was 0 points in 30/79 patients (38%), 1 point in 28/79 patients (35%), 2 points in 12/79 patients (15%), 3 points in 6/79 patients (8%), and 4 points in 3/79 patients (4%). Representative examples of imaging-based complications are shown in Fig. 3.

### Outcome

The median duration of follow-up was 13.3 months (IQR, 0.4–36.0). During follow-up, MACE occurred in 14/79 patients (18%). All events are summarised in Table 2. The median time between CMR and MACE was 10.2 months (IQR, 2.1–32.5). Events included re-hospitalisation due to acute symptoms (9/79, 11%) or due to chronic heart failure symptoms (4/79, 5%). Six out of 79 patients (8%) had new-onset arrhythmia (ventricular arrhythmia: 3/6 [50%], supraventricular arrhythmia: 2/6 [33%], third-degree atrioventricular block: 1/6 [17%]; see Table 2). Three of these patients subsequently underwent implantable cardioverter defibrillator (2/6, 33%) or pacemaker implantation (1/6, 17%). Two of 79 patients (3%) were diagnosed with a recurrence of TTS. Four of 79 patients (5%) passed away within the follow-up timeframe (one patient [1%] due to cardiac complication [cardiac arrest], two patients [3%] due to septic multiorgan failure, one patient [1%] had an uncertain cause of death). One patient (1%) experienced a stroke during the observational period.

**Table 2:** Events during the follow-up period of patients with Takotsubo syndrome

Clinical events	Patients (*n* = 79)
Re-hospitalisation due to new onset of acute cardiac symptoms	9/79 (11%)
New onset of arrythmia	6/9 (67%)
Torsades de pointes	1/6 (17%)
Ventricular fibrillation	2/6 (33%)
Inappropriate sinus tachycardia	1/6 (17%)
Third degree atrioventricular block	1/6 (17%)
Atrial fibrillation	1/6 (17%)
Implantable cardioverter defibrillator implantation	2/6 (33%)
Pacemaker implantation	1/6 (17%)
Recurrence of takotsubo syndrome	2/9 (22%)
Acute coronary syndrome	1/9 (11%)
Re-hospitalisation due to chronic heart failure symptoms	4/79 (5%)
Cardiac death	1/79 (1%)
Non-cardiac death	3/79 (4%)
All-cause mortality	4/79 (5%)

**Fig. 4:**
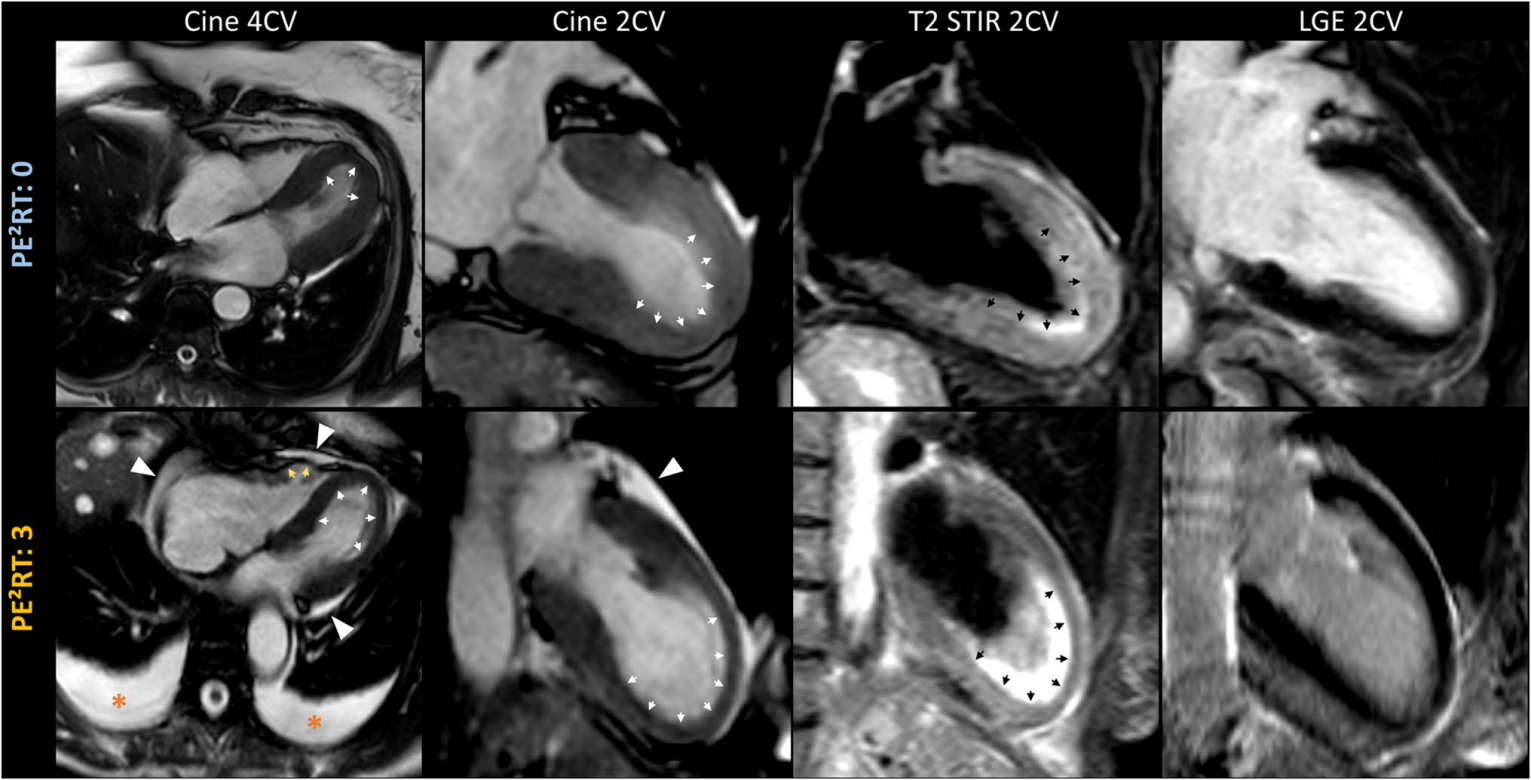
Representative cardiac magnetic resonance (CMR) imaging examples show two patients with typical diagnostic features of takotsubo syndrome (apical type). Regional wall motion abnormalities (apical ballooning) are visible on cine images in two- and four-chamber views (2CV, 4CV; white arrows) with corresponding myocardial oedema on T2-weighted short-tau inversion recovery images (T2 short-tau inversion recovery [STIR], black arrows) in the absence of late gadolinium enhancement (LGE). Images in the upper row show a 68-year-old female patient without major adverse cardiac event during follow-up (PE^2^RT score: 0; only slight pericardial fluid is visible on systolic cine images at the basal lateral wall, measuring  < 5 mm on cine images at diastolic phase). Images in the lower row show a 70-year-old female patient with the occurrence of a major adverse cardiac event during follow-up (PE^2^RT score: 3; moderate circumferential pericardial effusion  > 5 mm [white arrowheads], bilateral pleural effusions  > 20 mm [orange asterisks], and regional wall motion abnormalities involving the right ventricle [yellow arrows])

There were no significant differences in clinical and laboratory parameters between patients with and without MACE, except that of diabetes mellitus (Table 1). In patients with MACE, a physical trigger was more frequently observed (11/14, 79% vs 21/65, 32%; *p* = 0.001). On CMR, patients with MACE had lower LV ejection fractions (40 ± 15% vs 48 ± 12%; *p* = 0.03), higher heart rates (82 ± 19 bpm vs 70 ± 13 bpm, *p* = 0.004), and lower T2 signal intensity ratios (1.8 ± 0.3 vs 2.2 ± 0.5, *p* = 0.01). No significant differences were found in T1 and T2 relaxation times or ECV values (Table 1). Patients with MACE were more likely to present previously defined complications on CMR: pericardial effusion (12/14, 86% vs 22/65, 33%; *p* < 0.001), pleural effusion (10/14, 71% vs 15/65, 23%; *p* < 0.001), ventricular thrombus (4/14, 29% vs 3/65, 5%, *p* = 0.004), and RV involvement (8/14, 57% vs 9/65, 14%; *p* < 0.001). A representative clinical example is shown in Fig. 4. Accordingly, the PE^2^RT score was higher in patients with MACE (median [IQR], 2 [2,3] vs 1 [0–1]; *p* < 0.001). MACE did not occur in patients who did not have any of the PE^2^RT features (0/30, 0%).

**Fig. 5:**
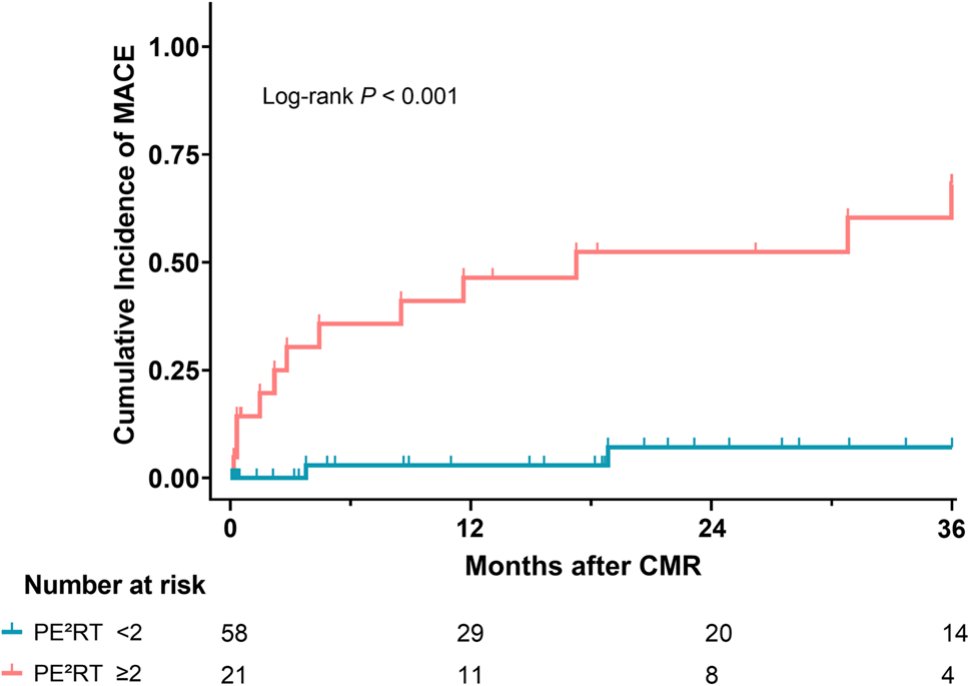
Kaplan-Meier curve analysis shows the difference in cumulative major adverse cardiac events (MACE) after patients were stratified according to the presence of two or more PE^2^RT features (pericardial effusion, pleural effusion, right ventricular involvement, ventricular thrombus) on cardiac magnetic resonance

In univariable Cox regression analyses, an association was found between the occurrence of MACE and physical TTS trigger, diabetes mellitus, heart rate, LV ejection fraction, T2 signal intensity ratio, pleural effusion, pericardial effusion, ventricular thrombus, and RV involvement (Table 3). Furthermore, an association was observed between MACE and the PE^2^RT score (HR per PE^2^RT point, 2.44; 95%CI: 1.62–3.68; *p* < 0.001; HR for PE^2^RT score  ≥ 2, 15.68; 95%CI: 3.51–70.10; *p* < 0.001). In multivariable Cox regression analysis, the presence of 2 or more PE^2^RT features was an independent predictor for MACE (HR for PE^2^RT score  ≥ 2, 7.98; 95%CI: 1.34–47.40; *p* = 0.02; see Table 4). Kaplan-Meier analysis showed that the presence of 2 or more PE^2^RT features was associated with the incidence of MACE (log-rank *p* <  0.001) (see Fig. 5).

**Table 3:** Univariable Cox regression analyses for the prediction of major adverse cardiac events (MACE) during follow-up of patients with takotsubo syndrome

	Univariable analysis	
Parameter	Hazard ratio	*p* value
Age (per year)	0.99 (0.95–1.02)	0.44
Sex (male)	0.73 (0.09–5.57)	0.76
Body mass index (per kg/m^2^)	1.06 (0.94–1.2)	0.36
Heart rate (per bpm)	1.04 (1.01–1.07)	**0.003**
Coronary heart disease	1.68 (0.54–5.24)	0.37
Atrial fibrillation	2.49 (0.54–11.38)	0.29
Arterial hypertension	5.59 (0.73–42.82)	0.10
Hyperlipidemia	1.29 (0.45–3.72)	0.64
Diabetes mellitus	3.35 (1.15–9.77)	**0.03**
White blood cell count (per 10^3^/µL)	1.07 (0.96–1.18)	0.23
Troponin I level (per ng/L)	0.83 (0.61–1.12)	0.22
Cardiogenic shock during hospitalisation	2.72 (0.59–12.43)	0.20
Intermediate care unit stay	1.91 (0.66–5.49)	0.23
Physical trigger	5.74 (1.59–20.77)	**0.01**
Emotional trigger	0.53 (0.12–2.36)	0.40
Non-apical ballooning	0.81 (0.18–3.64)	0.78
Left ventricular ejection fraction (per %)	0.95 (0.91–0.99)	**0.03**
Global longitudinal strain (per %)	0.98 (0.89–1.07)	0.63
Visible late gadolinium enhancement	0.70 (0.09–5.58)	0.73
T2 signal intensity ratio	0.18 (0.04–0.74)	**0.02**
Myocardial edema (focal vs diffuse)	1.64 (0.20–13.15)	0.64
Pleural effusion (> 20 mm)	4.81 (1.51–15.35)	**0.01**
Pericardial effusion (> 5 mm)	8.78 (1.96–39.42)	**0.005**
Ventricular thrombus	4.05 (1.26–12.96)	**0.02**
Right ventricular involvement	4.96 (1.71–14.34)	**0.003**
PE^2^RT score (per point)	2.44 (1.62–3.68)	** < 0.001**
PE^2^RT score (≥ 2)	15.68 (3.51–70.10)	** < 0.001**
PE^2^RT score (≥ 3)	5.50 (1.82–16.64)	**0.003**

Hazard ratios (95% confidence interval) are given. Bold values denote statisticial significance (*p* < 0.05).  PE^2^RT score was calculated by 1 point for each complication (pleural effusion, pericardial effusion, ventricular thrombus, right ventricular involvement)

**Table 4:** Multivariable Cox regression analysis for the prediction of major adverse cardiac events (MACE) during follow-up of patients with takotsubo syndrome

	Multivariable analysis	
Parameter	Hazard ratio	*p* value
Heart rate (per bpm)	1.00 (0.97–1.04)	0.89
Diabetes mellitus	2.85 (0.78–10.41)	0.11
Physical trigger	2.06 (0.50–8.39)	0.32
Left ventricular ejection fraction (per %)	0.99 (0.95–1.04)	0.76
T2 signal intensity ratio	0.52 (0.09–3.18)	0.48
PE^2^RT score (≥ 2)	7.98 (1.34–47.40)	**0.02**

Hazard ratios (95% confidence interval) are given. ﻿Bold values denote statisticial significance (*p* < 0.05). PE^2^RT score was calculated by 1 point for each complication (pleural effusion, pericardial effusion, ventricular thrombus, right ventricular involvement)

## Discussion

In this study, we demonstrated that easy-to-identify imaging markers on CMR summarised as PE^2^RT score (pericardial effusion, pleural effusion, RV involvement, ventricular thrombus) are frequently observed in TTS and are associated with MACE. At least one of these complications was observed in 62% of patients, and at least two of these complications were present in 27% of patients. A PE^2^RT score of  ≥ 2 was associated with MACE and was identified as an independent predictor in multivariable analysis. Our results demonstrate that a simple CMR assessment of concomitant complications may allow risk stratification of TTS.

In the past, TTS was believed to be a primarily benign syndrome due to recovery of LV function over the short to medium term in the majority of patients. However, in the last decade, several large multicentre studies have shown that the short- and long-term prognosis among patients with TTS is less favourable than previously thought [1, 2]. In a large registry study by Ghadri et al, the long-term outcome of TTS was comparable to matched cohorts of myocardial infarction or unstable angina [23]. In our study, the incidence of MACE was approximately 18% and all-cause mortality was about 5% during a median follow-up of 13.3 months. The event rates in our study are comparable to the study by Citro et al, which used a similar composite of MACE (overall MACE: 16.6%, overall mortality: 7.4%, median follow-up: 26.5 months) [24].

Several studies also demonstrated that different clinical (e.g., sex, age, physical trigger, cardiogenic shock) and imaging characteristics (e.g., LV ejection fraction, RV involvement, and non-apical ballooning) were factors related to worse long-term prognosis [1, 7, 8, 10, 25]. However, CMR data was not included in these outcome studies. In a recent study that was focused on the prognostic value of different CMR strain parameters in TTS, the longitudinal strain was identified as a valuable prognostic parameter; however, long-term mortality was mainly determined by comorbidities [26].

Although CMR is becoming more and more important for the diagnostic work-up of patients with suspected TTS [1, 12], its long-term prognostic value has not been sufficiently elucidated so far [16]. Complications, such as pleural or pericardial effusion, RV involvement, and ventricular thrombus, can all be easily assessed with standard CMR cine images without the need for quantitative analysis or time-consuming post-processing [27]. The frequency of these complications in our study (pericardial effusion: 43%, pleural effusion: 32%, RV involvement: 22%, ventricular thrombus: 9%) tends to be broadly consistent with previous CMR studies [12, 26]. In a CMR study of 34 TTS patients by Haghi et al, RV involvement was found in 26% of patients and was associated with lower LV ejection fraction and pleural effusion. The authors suggested that pathophysiological mechanisms caused by LV and/or RV dysfunction were responsible for this coincidence (elevation of pulmonary venous pressure and systemic venous pressure). Moreover, our results demonstrate that TTS patients who experienced MACE were more likely to have pericardial effusions, pleural effusions, RV involvement, and ventricular thrombus on initial CMR. A combined score based on these complications (summarised as PE^2^RT) revealed an HR of 2.38 per score point for the occurrence of MACE during follow-up. A PE^2^RT score of  ≥ 2 was associated with MACE and was identified as an independent predictor in multivariable analysis. These results are also in partial agreement with an echocardiographic study by Kagiyama et al that revealed a prognostic impact of RV involvement [10]. In a recent multicentre study, a clinical and echocardiographic-based score was developed for early risk stratification (in-hospital complications) of patients with TTS, which included male sex, history of neurologic disorder, RV involvement, and left ventricular ejection fraction [9]. Application of established prognostic scores, such as the CHA_2_DS_2_-VASc score and GRACE score (Global Registry of Acute Coronary Events), also allowed the prediction of mortality and MACE in patients with TTS [28, 29]. However, currently available risk scores for TTS often refer to early risk stratification, partly include parameters that may be more complex to measure and thus may differ between different clinical centres, and in particular, do not include CMR parameters.

LGE is an established marker of worse outcomes in non-ischemic cardiomyopathies; however, no associations were found between the presence of LGE and the occurrence of MACE in our study. This can be explained by the fact that TTS is predominately characterised by the absence of LGE [12, 18], although slight or patchy LGE might be detected using quantitative approaches [12, 30]. Since a visual approach was used in this study, LGE lesions primarily reflect coexisting pathologies (myocardial scarring/fibrosis), presumably without any causal relationship to TTS abnormalities [30]. Using centre-specific reference values, elevated T1 and T2 relaxation times were observed in more than 97% of patients with TTS, indicating acute myocardial injury/oedema. However, no difference was found between T1 and T2 relaxation times and MACE in the subgroup of patients with available mapping parameters.

Focal myocardial oedema correlating with typical RWMA is a major diagnostic criterion for TTS, which was present in 94% of patients in our study. Only 6% of patients had a primarily diffuse pattern of myocardial oedema, which could not be detected visually, but with the T2 signal intensity ratio or with T2 mapping. Patients with MACE, however, had less pronounced signs of diffuse myocardial oedema with lower T2 signal intensity ratio values, indicating limited reversibility of myocardial injury. The absence or a lesser degree of myocardial oedema has been reported to be associated with prolonged recovery time or worse outcomes in non-ischemic cardiomyopathies [31, 32].

Our study has limitations. Due to its retrospective design, some clinical and imaging data (e.g., myocardial mapping) was not available for analysis in every patient. Due to the relatively low number of events, the multivariable analysis might be prone to overfitting. Therefore, information on the incremental diagnostic value of the score is limited. Furthermore, our sample size was too small to generate a score that is based on a training and validation cohort. Therefore, the regression models are limited in their generalizability, and this study should be considered hypothesis generating. The proposed complication score needs to be validated in larger TTS cohorts and compared with other clinical and imaging parameters, preferably in prospective studies.

In conclusion, our CMR study shows that scoring four easy-to-assess complications (pericardial effusion, pleural effusion, RV involvement, ventricular thrombus) might be useful for long-term risk stratification among patients with TTS. All complications are relatively common in TTS and should generally be included in any CMR report, as their presence may also influence patient management (e.g., percutaneous puncture, therapeutic anticoagulation, or close patient monitoring). The proposed PE^2^RT score can be visually assessed using standard diagnostic CMR images and does not require any additional advanced quantitative analysis like strain analysis or mapping analysis and can therefore be applied in any clinical centre.

## Abbreviations

ACS: Acute coronary syndrome
CMR: Cardiac magnetic resonance
ECV: Extracellular volume fraction
IQR: Interquartile range
LGE: Late gadolinium enhancement
LV: Left ventricular
MACE: Major adverse cardiovascular events
RV: Right ventricular
RWMA: Regional wall motion abnormalities
STIR: Short-tau inversion-recovery
TTS: Takotsubo syndrome

## Declarations

### Guarantor

The scientific guarantor of this publication is Julian A. Luetkens.

### Statistics and biometry

L.W. (Institute of Medical Biometry, Informatics, and Epidemiology) kindly provided statistical advice for this manuscript.

### Methodology

• retrospective

• diagnostic or prognostic study

• performed at one institution
